# Portrait of a UK–Africa Capacity Building Initiative Consortium 2015–2022: the Cameroon, Ghana, South Africa and United Kingdom Materials Initiative (CaGSUMI) for developing materials for solar cells

**DOI:** 10.1098/rsfs.2023.0057

**Published:** 2024-08-09

**Authors:** Peter T. Ndifon, Johannes Awudza, Neerish Revaprasadu, Paul O'Brien, David J. Lewis

**Affiliations:** ^1^ Department of Inorganic Chemistry, University of Yaoundé I, Yaoundé, Cameroon; ^2^ Department of Chemistry, Kwame Nkrumah University of Science and Technology (KNUST), Kumasi, Ghana; ^3^ Department of Chemistry, University of Zululand, Private Bag X1001, KwaDlangezwa 3880, South Africa; ^4^ Department of Materials, University of Manchester, Oxford Road, Manchester M13 9PL, UK

**Keywords:** materials, UK–Africa, Cameroon, Ghana, South Africa

## Abstract

The CaGSUMI consortium was funded by the Royal Society–Department for International Development (later the Foreign, Commonwealth & Development Office) on the Africa Capacity Building Initiative programme between the years 2015 and 2022 and involved three Sub-Saharan African universities: Kwame Nkrumah University of Science and Technology, Kumasi, Ghana, University of Yaoundé I, Cameroon, and the University of Zululand, South Africa; and the University of Manchester in the United Kingdom. The project was used to cement an emergent UK–Africa network in the areas of materials chemistry related to renewable energy generation with both thin films and nanomaterials. The consortium’s outputs led to numerous publications of African science in international journals, a number of graduated PhDs who went on to permanent academic positions and prestigious fellowships, the establishment of a capacity-building plan relevant to the chemistry departments in each of the African countries, and the installation of a number of first-in-kind pieces of kit for African laboratories that will keep them on a competitive footing at an international level for the next decade and more.

## 1. Introduction: genesis of a consortium

There had been several informal interactions over the years from the original PI of the consortium, Prof. Paul O’Brien FRS CBE (d. 2018), with groups in Sub-Saharan Africa, including those in Ghana led by Prof. Awudza, Prof. Ndifon and Prof. Revaprasadu—the latter academic was Prof O’Brien’s PhD student and hence strong working links were already in place. There had also been funded interactions outside of this. For example, O’Brien had been the holder of three Royal Society (RS)–Leverhulme grants: two with Prof. Egid Mbofu (d. 2018) in Tanzania, and one at the time of the application that was running with Awudza in Ghana. On top of this, O’Brien had also led a Royal Society South Africa programme between the years 1996 and 2008 that had focused on capacity-building work in post-apartheid South Africa. Officially, CaGSUMI was the first project in which all four partners worked together. As part of the implementation of this project, a number of workshops were organized at the Kwame Nkrumah University of Science and Technology (KNUST) with all four partners contributing equally to draft the research programme. All partners were then invited to participate in the Royal Society’s preparatory workshop for bidding for the RS–Department for International Development (DFID) project in 2013 in Dakar, Senegal. Following that, a Network Grant worth £25 000 was awarded to the proto-consortium by the RS–DFID programme and this was used to develop the network for the CaGSUMI project. Also around the time of the application, the University of Manchester (UoM) had introduced its new social responsibility agenda that the DFID Africa Capacity Building was fully aligned with. For all partners, there was considerable stakeholder interest in receiving funding for such a programme; clearly, by this time, there was considerable momentum in the research based on both historical funding and the organic collaborations that had grown over the years.

## 2. The African partner institutions

In December 2014, the original four partners were informed that they had been funded by over £1 million by RS–DFID for 5 years for the new RS–DFID Africa Capacity Building Initiative (ACBI) programme. This was the largest award so far for the consortium and would be one to achieve the lasting and transformative impact in Sub-Saharan Africa that O’Brien, in particular, had been striving for over his career.

The University of Zululand (UNIZULU) was established in 1960 as the University College of Zululand, a constituent college academically affiliated to the University of South Africa. In 1970, UNIZULU attained university status. UNIZULU gained full autonomy in 1984. In 2002, the university was declared to be the only institution of higher learning north of the Thukela River and began to include outcomes-based programmes in its curriculum. After this time, the university experienced an increased intake of students from other parts of Africa, especially from Namibia, Nigeria, Kenya, Zimbabwe, Botswana, Lesotho and Swaziland. The Overarching Reconfiguration Committee was established to represent all stakeholder groupings on campus as well as local businesses so as to generate policies and principles to facilitate the goal of reconfiguring the university into a Comprehensive Institution, as decreed by the Department of Education in May 2002. Historically, UNIZULU was classified as a Historically Disadvantaged Institution (HDI) considering policies established during the apartheid era in South Africa. Although the institution is now reclassified as a comprehensive institution and no longer operates as an HDI, there remains a post-apartheid legacy. Decades of neglect in addressing the higher education needs of the majority population because of apartheid policies created a myriad of challenges that UNIZULU is committed to addressing in its contribution to the shaping of the new South Africa. UNIZULU had an active materials research programme prior to the award, and on paper was the most developed of the three African partners in terms of its capacity for research. This was initiated through the previous NRF/RS capacity-building programme which ran from 1996 to 2005. Professor Revaprasadu was awarded the prestigious SARChI Chair in Nanotechnology in 2007. UNIZULU also had characterization equipment purchased through these programmes which included a powder X-ray diffractometer as well as a range of spectrophotometers (e.g. UV–vis, FTIR) and scanning probe instruments (e.g. AFM).

The University of Yaoundé I (UY) was created in October 1961 as the Institute for University Studies (l’Institut d’Etudes Universitaires). It was built with the help of France and opened in October 1962 as the Federal University of Yaoundé, dropping the ‘Federal’ in 1972 to become the University of Yaoundé. Before the university reforms of 1993, the University of Yaoundé was experiencing an explosion in student numbers. With a student population of more than 40 000 on a campus built to accommodate less than half that number, it was split into two: University of Yaoundé I and University of Yaoundé II. The main mission of the university is to provide opportunities for quality education through teaching and research. The university is dedicated to the reinforcement of professionalization by promoting the relevance of curricula in order to meet labour market requirements. Its teaching and research programmes are also aimed at supporting socio-economic and cultural development, the practice of bilingualism, the promotion of moral and human values and service to the community. UY is a bilingual institution (French/English) with five faculties/schools, four specialized centres with a student population of 46 000 served by 1042 teachers. The governance system of the university basically follows the French system with some British influences. The university offers a variety of degree programmes at the Bachelor’s, Master’s and PhD levels, all of them designed to comply with the European Bologna LMD/BMP System. At UY prior to the award, there existed some undergraduate and postgraduate programmes on materials chemistry including composites and nanocomposites, ceramics, coordination chemistry, nano-channelled materials, etc., but UY depended on international collaborators for materials characterization.

**Figure 1.**
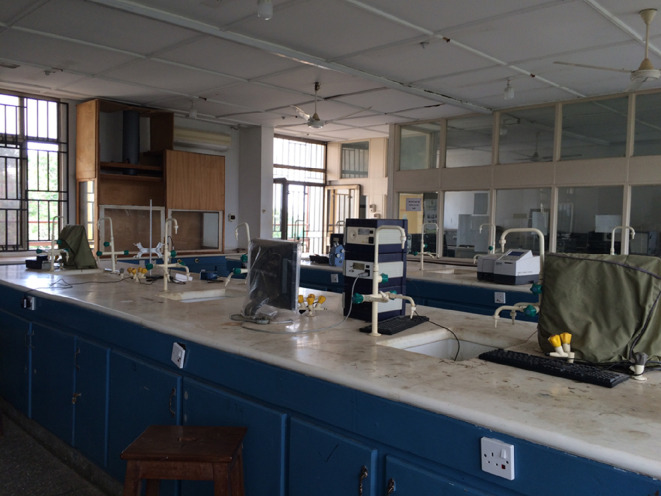
Typical wet chemistry laboratory at KNUST, equipped with fume cupboards.

KNUST was established in 1952 as the Kumasi College of Technology and became a fully fledged university in 1961. It is the premier science and technology university in Ghana, producing most of the graduates in these fields in the country. It has the vision of being globally recognized as a premier centre of excellence in Africa for teaching and research in science and technology for development; producing high-calibre graduates with knowledge and expertise to support the industrial and socio-economic development of Ghana and Africa. KNUST also offers services to the community, is open to all the people of Ghana and is positioned to attract scholars, industrialists and entrepreneurs from Africa and other international communities. The current student population (2022/2023 academic year) is 89 047, made up of 53 693 (60%) males and 35 354 (40%) females, and it is working hard to increase the number of female students and staff (academic and non-academic). A total of 58.1% of the courses offered at KNUST are STEM based. A picture of a typical chemistry laboratory at KNUST is shown in figure 1. Prior to the programme, a Royal Society–Leverulme Trust Project (2010–2014) was running at KNUST which was being used to develop solar cell materials prior to the CaGSUMI project. Under this project, three people obtained their PhDs: Prof. Francis Ampong (thesis: ‘Synthesis and characterization of CdZnS by chemical bath technique’), Prof. Noah Asare-Donkor (thesis: ‘Structure and optoelectronic properties of copper, zinc, cadmium and lead chalcogenide nanocrystalline thin films deposited at the water–toluene interface’) and Prof. Nathaniel Owusu Boadi (thesis: ‘Synthesis of lead chalcogenide nanoparticles and thin films from single-source precursors’). In addition, one person (Dr Selina Ama Saah) obtained her MPhil on characterization of lead sulfide nanoparticles from lead alkyl xanthate single-source precursors using a solventless method. In addition, KNUST was carrying out some work on polymer-based composites and materials under a Development Partnership in Higher Education (DelPHE) project (supported by the British Council) which we were running with the School of Chemistry of the University of Manchester. The Royal Society–Leverhulme Trust Project also allowed KNUST to acquire equipment to measure photoluminescence, UV–visible absorption and infrared spectra for characterization.

## 3. The CaGSUMI research and training programme

The four research groups in the consortium had interests in directing materials chemistry approaches to producing materials for energy generation—in particular earth-abundant metal chalcogenides [1] that would allow African nations to have access to energy materials to establish their own energy security. Each group had their own angle of attack on the problem; O’Brien’s research had been focused for some time on the development of precursor-led routes towards quantum dots for energy generation (e.g. CdTe) [2] and he had recently spun out a company (Nanoco) in this technological area. The O’Brien laboratory, in particular, had access to the most cutting-edge characterization techniques at the University of Manchester (e.g. the newly installed aberration-corrected Titan ChemiSTEM) that would allow a step change for the African laboratories in characterization of their materials, and new insights into performance. Revaprasadu’s laboratory was focused on developing narrow-gap semiconductor materials [3–5] that could also be useful for applications beyond solar energy, for example, in water splitting to produce H_2_ and O_2_ from H_2_O. Ndifon’s group was a more traditional inorganic coordination chemistry group that would bring further expertise in developing new and novel precursors to unlock chemical routes to new inorganic materials [6]. Awudza’s group brought the novel aspect of using natural products as agents to control nanocrystalline growth and represented the sustainability aspect of the project [7–9]. The original aims of the project were to:

— make significant progress in developing routes to materials that are useful in solar cell applications, and in doing so, to create a sustainable network of scientists in Sub-Saharan Africa working in materials chemistry;
— engender student training in materials synthesis, characterization and related professional skills;
— develop research infrastructure at African institutions; and
— build and strengthen research capacity at African institutions.

The more than eight PhD projects were designed with travel in mind to maximize the exposure of the students to these aspects of the laboratories so that they could tailor and develop their PhDs towards their own interests. This was felt by all PIs to be the correct approach for training independent thinking and allowing the students to develop their own ideas for independent projects after the programme to engender lasting impact.

The PhD topics included the following:

UNIZULU

PhD 1: Thiosemicarbazone, Xanthate and Dithiocarbamate Single Source Precursors for Cadmium, Lead and Indium Sulfide Nanoparticles.

PhD 2: Novel Precursors for the Synthesis of Metal Selenide Nanoparticles, Thin Films and Alloys.

PhD 3: Chalcogenocarboxylate Complexes for the Synthesis of the Corresponding Binary, Ternary and Quaternary Nanomaterials for Energy Applications.

UY

PhD 4: Metal Complexes of Thioureas, Dithiocarbamates and Xanthates as Versatile Molecular Source Precursors for the Fabrication of Metal Sulfide MS (M = Pb, Cu, Cd, In and Ga) Nanoparticles and Thin Films.

PhD 5: Manganese Sulfide and Iron Sulfide Undoped and Doped Nanomaterials from Ethylpiperazine Dithiocarbamate Complexes as Single Source Precursors: Synthesis, Characterization and Optical Properties for Energy Applications.

PhD 6: Synthesis, Characterization and Applications of some Bismuth(III) and Antimony(III) Sulfide Nanomaterials.

KNUST

PhD 7: Green Approach to Metal Chalcogenides Semiconductor Nanomaterials: Syntheses and Characterization of CdS, PbS, ZnS, MnS and MnZnS Nanoparticles.

PhD 8: Synthesis and Characterization of Bismuth and Lead Chalcogenides and their Ternary Alloys from Single-Source Precursors.

PhD 9: Dye-Sensitized Solar Cell Materials Using Local and Natural Raw Materials for the Preparation of the Dyes.

**Figure 2.**
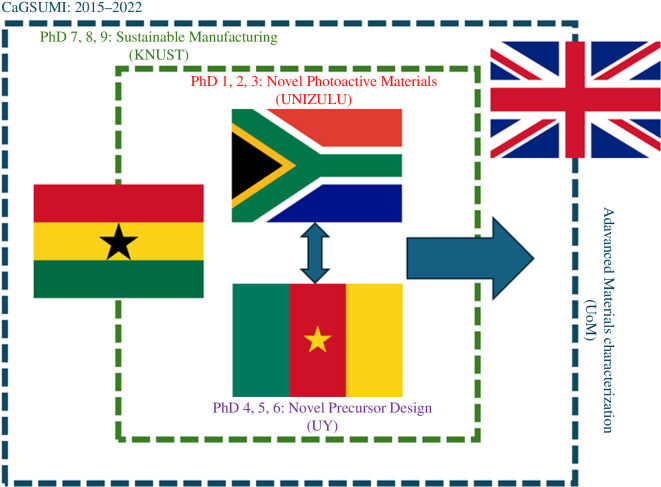
Operationalization of PhDs within CaGSUMI and the relationship to each other.

These PhDs were operationalized into a structured and coherent programme with UNIZULU and UY leading the novel precursors/materials aspects, KNUST investigating the sustainability of the manufacturing processes used and UoM providing advanced materials characterization for all partners, based on students visiting and analysing their own samples while being trained on instruments not available at their home institutions, yet taken for granted as routine analysis in the European setting (figure 2).

**Figure 3.**
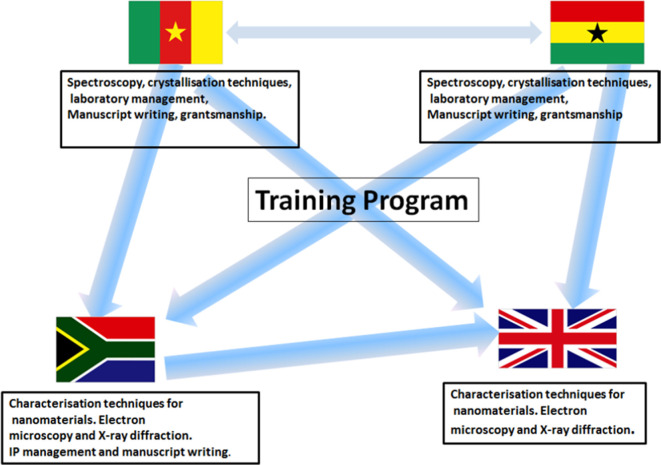
CaGSUMI training programme, covering both academic skills and skills useful for agency in research. Each box represents agenda items held at the planned training workshops in the respective countries. As it turned out, some of these were impossible to host due a range of *force majeure* events including political instabilities in Cameroon, the death of the original PI Prof. O’Brien FRS and the COVID-19 pandemic; only the training workshops in South Africa and Ghana were implemented.

All projects were linked to a training programme that was held in rotation at each of the sites annually for the students, which covered both academic aspects of the materials chemistry (e.g. crystallography, electron microscopy, characterization techniques, air-sensitive preparation techniques; figure 3) but also management and writing skills useful for their further careers (e.g. grantsmanship, IP management, laboratory management). There was also training delivered outside of these aspects to staff at each of the universities including lectures on safety attended by students and staff of the whole department. A summary of the planned training programme for the materials chemistry PhD students and group associates on the project is shown in figure 3. The training aspects were delivered by the principal investigators on the projects, but also postdoctoral researchers from each of the laboratories. It, however, must be noted that owing to a number of *force majeure* events, e.g. the unexpected and premature death of Paul O’Brien in 2018, the political unrest in Cameroon in 2019–2020, and the COVID pandemic in 2020–2022, the training programme was not delivered onsite in the UK or Cameroon during these periods. However, the money saved from not hosting these events face-to-face was employed very successfully elsewhere and was to provide a step change in research capacity building later in the project (vide infra).

## 4. Development of a research capacity-building plan

One of the key aspects of the project that made it very different from traditional granting based on the funding of research alone was that there was the requirement during the programme to develop a capacity-strengthening action plan for the PhD programmes [10], tailored to the needs of the CaGSUMI institutions. We were not aware that such an exercise had ever been undertaken at any of the institutions prior to this. Therefore, this exercise was one of the most important aspects of the project, with the aim to give the African partner institutions a plan of action on a number of fronts by which to improve the academic and research environment for their staff and students, and the design of the plan was made over the period 2018–2019 and is shown in table 1. In developing the plan for CaGSUMI, the consortium had meetings with the group of Professor Imelda Bates and Dr Taghreed El Hajj from Liverpool School of Tropical Medicine with existing literature on the topic being taken into account as a starting point [10–13]. The plan was developed with input from all consortium partners to ensure a broad approach that is applicable to other science departments across Sub-Saharan Africa if deployed elsewhere outside of the CaGSUMI programme. Professor Bates (vide infra) encouraged all consortium members to share this plan with key stakeholders in their faculty, particularly with senior members with the agency to enact change across institutions.

**Table 1.** The CaGSUMI capacity-building plan developed with input from colleagues at Liverpool School of Tropical Medicine (Prof. I. Bates, Dr T. El Hajj).

goal:	to produce doctoral programmes that are aligned to UK/EU programmes in terms of their structure and research environment, where top-quality candidates can perform world-leading research							
institution	capacity gap	activity to fill the gap	indicator/evidence	funding needed	person responsible	expected date of completion	progress made	comments/explanations
1. Institutional policies and structure								
objective: produce PhD programmes that have adequate policy and institutional support								
African universities	lack of comprehensive policies for management of PhD programmes	comprehensive policy documents to be developed which outline institutional regulations governing PhD programmes	comprehensive policy documents are available to download by staff and students	no external resources needed	faculty PGR coordinator; head of department	2 years		Yaoundé University is bilingual; these documents must be produced in both French and English
African universities	lack of student handbook	faculty to produce a handbook detailing course structure and expectations	comprehensive course handbook is available to download by staff and students	no external resources needed	faculty PGR coordinator; head of department	2 years		Yaoundé University is bilingual; these documents have to be produced in both French and English
African universities	lack of professional support services for IT, library, admin, finance	recruitment of staff in these areas in line with expansion in doctoral training programmes	staff recruited committee meeting minutes available	external resource may be needed	dean of faculty; head of faculty; professional support; head of department	5–10 years		financial reconciliation this year highlighted a lack of professional support in finance in at least two of the institutions in the consortium; the academic often has to do the accounting themselves
2. Research environment								
objective: to engender a safe, collaborative research environment with the necessary facilities to perform independent world-leading research								
African universities and UK institution	access to electronic resources including expensive software and journals located behind expensive paywalls	access through UK institutions and collaborators; longer term: provision must be made for these in budgeting	student satisfaction survey/exit interviews	no external resources needed short term; longer term, institutions should be lobbied by academic staff to budget for access	chief librarian; head of department	2 years		access to primary literature can accelerate research programmes to competitive levels
African universities	no learning/meeting space for students and poor Internet connections	produce a dedicated ‘learning commons’ with broadband internet connection and PC workstations/access points	space is available; student satisfaction survey/exit interviews	no external resources needed	dean of faculty; head of department	2 years		learning commons can encourage new collaboration, improve, Internet connectivity to enable larger literature searching and capacity for online meetings (e.g. via Skype, Teams, Zoom)
African universities	inadequate laboratory space and facilities for top-level materials research	refurbish laboratories	laboratories refurbished	external	dean of faculty; head of department	10 years	Zululand laboratories were set to be refurbished in 2018	includes inability to handle air-sensitive materials, no electron microscopy, lack of fume hoods, no temperature- and humidity-controlled spaces. All of these are crucial to top-tier materials research
African universities	regular student staff briefings and seminars	arrange monthly briefings and seminars by academic staff to students or vice versa within the section	publication of a list of speakers for the semester; meetings arranged and delivered	no external funding needed but may require a significant amount of internal resource to develop and maintain	head of section	1 year		a regular seminar series can broaden research horizons, encourage new collaborations. Can also be a good opportunity for students to present their research
African universities	lack of robust safety culture	introduction of a safety ‘workflow’ and paper trail. Leaders to adopt SAFER leadership principles (Wong, Kelloway and Makhan, 2016). Each research group is to nominate a safety champion and regular are meetings held	introduction of CRA and COSHH and evidence of usage. Regular departmental safety inductions and refreshers. Introduction of accident and near-miss reporting. Safety committee meetings	no external funding needed but may require a significant amount of internal resource to develop and maintain	dean of faculty; head of department; safety champions/officer	3 years		suggest a UK/EU style system as a paradigm. Could be enhanced by creating dedicated positions for safety officers with attractive salaries and career progression
African universities	prioritizing research areas to produce high-impact work and attract funding	formation of a departmental research strategy committee. Attendance of staff at international conferences. Engagement of policymakers and external funders as well as potential research stakeholders to identify priority areas that may attract funding	research committee meeting minutes. Staff attendance at conferences. Increased external funding from a range of sources	no external resources needed	head of department	3 years		
3. Recruitment and admissions								
objective: to provide systems for efficient and effective recruitment, and to provide a comprehensive induction for PhD candidates who have funding secured to complete their studies								
African universities	robust interview process during recruitment and checking of grades and for international students IELTs, etc., and complying with EDI principles	two members of staff interview prospective PhD students to ensure quality. Minimum standards of English language for recruitment. Training in principles of EDI	policy on recruiting students updated	no external resources needed	head of faculty; head of faculty HR	3 years		could mirror UK student recruitment policy as paradigm
African universities	formal induction for students	introduce formal departmental induction for every student and new starter	induction timetable published and referenced in programme handbook	no external resources needed	head of department	2 years		
African universities	ringfenced funding for PhD students	all programmes are to be fully funded from start to finish with assurances given in writing to students prior to starting	contracts and offer letters to individuals affirming funding covered for duration of study. Departmental budgets and financial statements. Exit interviews/student satisfaction surveys	university funds to faculty or department. Stipends from external funding bodies including industry and research council equivalents	head of faculty; head of faculty HR; external funding contacts	2 years		
4. Quality PhD programmes—supervision, training, assessment and appeals								
objective: to produce rigorous, robust, accessible PhD programmes that allow students to continually improve their skills, which are consistent in assessment and with recourse to independent mitigation and appeals in cases of non-progression								
African universities/UK university	ensuring quality supervision	workshops on supervisory skills, research ethics. Regular mentoring for supervisors by senior staff or senior UK mentors. Nomination of a second supervisor to ensure quality. Roles and responsibilities outlined formally at start of programme including the agreement on IP position and publication roles	workshop attendance. Evidence of active mentoring programme/joint supervision of students. Signed documentation agreeing roles and responsibilities	no external funding needed	PIs; PGR supervisor	3 years		mentoring is a key part of the UK academic system and should ensure honest appraisal and performance of staff. Mentoring systems can be risky owing to cronyism and favouritism, fear of reprisal and hence staff from different faculties should be considered for mentors
African universities	student professional skills development	formal opportunities for training are offered in a broad range of professional skills related to science careers	training catalogue available to students. Evidence of attendance of courses offered. exit interviews and student satisfaction	no external funding needed but may require a significant amount of internal resource to develop and maintain	head of department	2 years		Bates *et al.* [10] suggest making training a compulsory part of gaining credit in doctoral programmes as a requirement of progression
African universities	consistent assessment of doctoral programmes	progress and completion to be monitored in doctoral programmes to ensure consistency. Substantial independent examiners report for candidates as well as joint examination report from viva. Exam boards to be set up that review examination reports and investigate and address non-progression in all cases	5 year progression statistics available on demand. robust procedures in place for selection of examiners as well as examination reporting. Exam board reports and minutes	no external funding needed. Internal resource staff time	head of faculty	2 years		
African universities	robust mitigation and appeals procedures	robust and independent mitigation and appeals procedures to be introduced with standard proformas and evidence requirements for each, and handled by staff independent to the PI in all cases	information where to seek appeals and mitigation included in programme handbook. Departmental academic-led student welfare lead position created	no external funding needed	head of faculty	2 years		
African universities	provision for disabled students	set up a Disability Advisory Support Service (DASS) for disabled students that can offer support for studying	information included in programme handbook	some external funding needed and may require a significant amount of internal resource to develop and maintain	university	5 years		*ca* 15% of students register with disabilities at UoM; Manchester has a DASS service that outlines typical services provided (http://www.dass.manchester.ac.uk/)
5. Feedback and evaluation								
objective: to create a feedback loop where feedback and evaluation is used to continually improve doctoral programmes								
African universities	feedback on programmes	exit interviews/student satisfaction survey. Progress and completion rates	annual review meeting minutes	no external funding needed	head of department	2 years		
African universities	external evaluation of programmes	consider appointment of external examiners that can formally appraise doctoral programmes annually	external examiners reports	payroll for external examiners needed	head of faculty	2 years		
African universities	proactive capacity building	monthly capacity-building committee formed to address faculty-wide capacity-building activities	monthly meeting minutes	no external funding needed	head of faculty	2 years		

The plan focused on a number of key areas and was focused squarely on the PhD programmes within the institutions.

1. *Policy and structure*

These included capacity-strengthening plans to introduce PhD programmes that have adequate policy and institutional support. Ideas suggested where institutions could improve revolved around the development of policy for the management of PhD programmes, and subsequent development of these documents, and often the lack of a student handbook to aid induction into the PhD programmes at each institution. The plan also highlighted areas such as the lack of institutional support in areas like IT, which are so crucial to modern research programmes for analysis and storage of data.

2. *Research environment*

This part of the plan was devised with the objective of engendering safe, collaborative research environments with the necessary facilities to perform independent world-leading research. Granular areas within this section of the plan included ensuring access to electronic resources, creating meeting spaces with Wi-Fi for students with often poor Internet connections outside of the university, laboratory refurbishment programmes and regular staff–student briefings on the plans for research at the institutions including staff seminars. The lack of robust safety culture was also identified as an area that could be very much strengthened in all the institutions, with leaders adopting the principles of SAFER leadership, and leading safety culture from the front. The production of research strategy documents by which to horizon scan and identify areas where funding may become abundant in the future was also part of the plan within this section.

3. *Recruitment and admissions*

The plan included objectives to provide systems for efficient and effective recruitment, and to provide a comprehensive induction for PhD candidates who have funding secured to complete their studies. The improvement of the quality of candidates for the programmes long term will result in the improvement of research; robust interviewing processes need to be implemented across the board that comply with modern EDIA principles to create talented and diverse research teams. A departmental induction should be offered and there should be ringfenced funding in budgets for the provision of PhD students to (especially new starter) groups.

4. *Developing quality PhD programmes*

The focus of this part of the plan was to provide a robust process for supervision, training and assessment in PhD programmes. It also included plans for introducing appeals processes for PhD examinations, and, importantly, the setting up of a disability advisory and support (DASS) service at African institutions.

5. *Feedback on PhD programmes*

The plan also covers feedback elements of PhD programmes, which included proposals for the introduction of exit interviews, for the appointment of external examiners and a capacity-building committee at each institution to be established so that there is accountability for capacity across departments, schools and faculties and colleges.

## 5. Challenges during the programme

One of the first major challenges that the consortium encountered was the political instability and civil unrest in Cameroon (since 2017), that brought the country to the brink of civil war in 2018. This precluded the running of a training workshop for staff and students at that institution. The year when we planned to go to Cameroon subsequently (2020) was impacted by COVID. Hence these external factors meant that we could not achieve equitable training on the programme which was very unfortunate.

**Figure 4.**
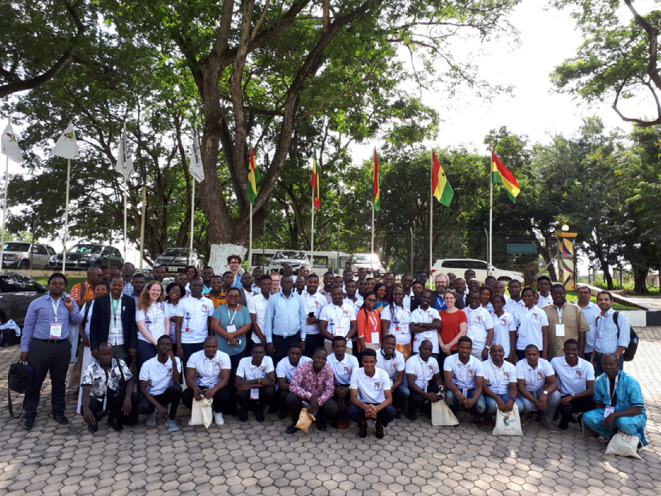
Consortium meeting in Ghana 2019. Unbeknown to us, this would be our last face-to-face meeting of the CaGSUMI Consortium and associates.

The second major challenge that the programme experienced was the untimely death of Paul O’Brien from glioblastoma multiforme. Paul had become unwell in January 2018. The RS visited UoM in late spring 2018 to invite David J. Lewis to substitute for Paul, who was growing more and more ill and was unable to work on the project, with the latter’s blessing; Lewis had been a postdoctoral researcher and a research fellow in the group of O’Brien at Manchester Chemistry but was at that time an independent lecturer in the Department of Materials. While unable to replace the irreplaceable it at least gave a steady hand on the tiller. A meeting in August 2018 was held in Manchester as Paul’s health deteriorated, which included the chance for the African PIs to visit their friend, for what would be one last time. Paul died peacefully at home surrounded by his family on 16 October 2018. Much has been written about his life and work since, and particularly on his work in Africa. The untimely death of the PI in service significantly delayed aspects of the programme (e.g. visits to UoM were mainly impossible for the first six months of 2018); the lack of visits by African students to UoM, in particular, was destructive to the planned programme; for many students, this had been critical in the publication of their work, and many of the leaders and students also lost their close friend which also impacted their mental well-being. Anyone who had worked with Paul will understand how he was in many ways the driving force behind these programmes and the gel that held much of the consortium together. The psychological effect on all the researchers on the programme of course cannot be documented but objectively this was a devastating blow to the team’s morale, and at some points in 2018 it seemed that the network would collapse entirely. However, at our meeting in August, we had decided we had to continue the programme primarily because this is what Paul wanted; the meeting was also critical to pick up the plans, briefing the new UK PI, and to define how we would approach the programme in Paul’s absence as PI—we recognized that things would be much different from before, and perhaps harder without Paul’s knowledge, boundless enthusiasm and generosity, but we also recognized that the programme was of key importance for the African institutions and should continue in earnest. By speaking to each other frankly and face to face and having everyone in the room this was very clear to see. By 2019, the programme had re-established visits to each other’s laboratories, and a consortium management and training meeting was held at KNUST in Ghana in March, with a satellite international materials conference attached to this (figure 4). The satellite conference was dedicated to the memory of Paul O’Brien. It was felt by all that the next few years would be far simpler in comparison with the trauma we had just been through. How wrong we were.

The COVID-19 pandemic began in March 2020. For all the universities involved in the consortium, this would quickly turn to their governments locking down for many months in the first wave, and again for subsequent waves of the virus, now endemic in all four societies. This had a major effect on the consortium—primarily that students could not travel in person, which had been the main *modus operandi* for the collaborative research together from the start of the programme. The consortium leaders met online in early summer 2020, for the first time via Zoom, to establish an emergency plan of action for the year.

From this meeting, several important points were decided that would shape the direction of the consortium in its final 2 years:

— The analysis of samples would continue through 2020 but by mailing samples and with no physical visits.
— Consortium meetings and the annual meeting were to be held online until further notice—for the annual meeting this turned out to be a marquee online materials chemistry conference—*Precursor Chemistry for Materials Synthesis*, and allowed the students on the programme to present their work alongside research leaders in the area (e.g. Prof. Brutchey, University of Southern California; Prof. Sir Richard Catlow FRS, UCL/Cardiff University; Prof. McElwee White, University of Florida; Prof. Carmalt, UCL), many of whom we probably would not have been able to afford to invite had we held the conference face to face. For the regular consortium meetings, it meant that we probably spoke more often than if we would have continued with face-to-face meetings.
— The consortium would look into converting most (>90%) of its remaining, and significant, travel budget into the purchase of materials before the end of the programme with the view that this would allow the African partners to complete the research objectives of the grant without significant travel to Manchester, but would also mean that there would be lasting impact at the African institutions beyond the lifespan of the ACBI programme. We planned capital equipment purchases to spend this part of the budget now.

## 6. COVID-19 brings opportunity: the purchase and delivery of capital research equipment for the African universities

As mentioned in the previous section, the COVID-19 pandemic prevented all international travel during 2020–2021 as the search for a vaccine went ahead so that societies could unlock again. Lewis approached the Royal Society in late summer 2021, backed by the other members of the consortium, to explore the idea of re-purposing the significant remaining travel budget into the purchase of capital items of equipment. It had been identified for example that with X-ray diffractometers to Ghana and Cameroon, that the students would be able to complete their projects there. The original ACBI guidelines had capped the spend on equipment to only small pieces of equipment. Lewis was asked by RS to provide a proposal to justify the equipment which was written, submitted, reviewed and accepted by RS in December 2021. A capital expenditure case was made to UoM in January 2022 which was approved by the Dean of the Faculty and the purchase process began in earnest in February/March 2022. Our strategy for purchasing was to aim for manufacturers to deliver instruments direct to the African nations. Previously, we had not used this approach—we had directed all purchases through UoM and organized and paid for separate postage to the African universities for the small equipment in 2015/2016. In general, we worked hard to ensure the quotes for capital equipment included delivery to local ports or airports close to the African universities. We found that in many cases, the large organizations we purchased from had a network of local distributors that were used for local deliveries. In addition to this, universities were able to reach agreements with ports and airports that avoided hefty taxes being applied on delivery. All capital instruments were delivered by the end of the ACBI programme.

## 7. Outputs and successes from the programme and beyond

The research in the programme graduated nine PhDs in materials science, and led to the publication of over 30 papers with both directly funded and associate students aligned to the project as lead authors [6,8,9,14–43], and was one of the most successful consortia on the RS–Foreign, Commonwealth & Development Office (FCDO) programme in terms of academic research outputs. In a recent RS peer review of the ACBI programme in 2023, the outputs from our consortium on average were rated at 5* (very good).The consortium had 10 papers assessed by two reviewers, with 9 of the 10 papers receiving a score of 4 (good) or above. Paper 1 (Kun *et al*. [15]) was highlighted as being particularly strong, with one reviewer commenting that it was ‘a strong paper with good interpretation and explanations of results. Data is well presented and discussed’. More generally, the reviewers considered that the papers presented impactful research, with significant and detailed results. The novelty and creativity of some of the outputs from our consortium were noted. For some outputs, the analytical techniques were considered relatively limited (this is, of course, to be expected), but importantly now ‘appropriate’, and data were stated to be well presented and discussed throughout. New classes of solar energy generation materials were developed across the board.

One female PhD student from KNUST secured a permanent academic position at another institution in Ghana, while a female research associate who was staff at UY on the project also obtained a permanent position at the same institution. A male PhD student from KNUST won a prestigious Royal Society Leverhulme Africa Fellowship in 2018 to branch out into nanomaterials for water remediation [44]. A female researcher obtained an O–SD—Early Career Women Scientists (ECWS) award. Through this award, a training seminar, ‘OWSD Nano Days 2023: A Two Days Training Workshop’, was organized in the faculty of science in 2023. UY also obtained some equipment, including a photoreactor and reagents through this programme.

The PIs on the grant were also successful with career awards and promotions, many arising owing to work on the programme; O’Brien himself was awarded a CBE in the Queen’s New Year’s Honours List in 2016 for his services to science and engineering, and was made Fellow of the Royal Society of Engineering in the same year. Professor Revaprasadu was elected a Fellow of the Royal Society of Chemistry in 2021. He also received a NRF B-2 rating in 2023. The rating means that all or the overriding majority of reviewers are firmly convinced that the applicant enjoys considerable international recognition for the high quality and impact of his/her recent research outputs. Dr Malik Dilshad Khan also received a NRF Y-2 rating in 2020. The UNIZULU research group is now well known both nationally and internationally for developing functional materials for applications in energy. Lewis was made Professor of Materials Chemistry at Manchester and Fellow of the Institute of Materials, Minerals and Mining in 2023. He took over the role of Head of Department at Manchester Materials in 2024. The leadership skills acquired on this grant were key to these career successes for him.

The grant was aimed squarely at producing equitable partnerships in terms of the subject area that it explored; the energy security for African nations is currently a challenge in equity between those places and more energy-secure areas like Europe. The nature of the travel and the even monetary distribution between partners also ensured that there was equity between the partners in more resource-poor settings. To address equity in the ethics of research (which we realize has different cultural approaches and therefore diverse views) training was built into the programme, with some focused on ethics in research as it is perceived in UK institutions. The visits to other institutions in the network by both consortium members and PhD students facilitated interactions between us and non-consortium partners (resource persons for training). Students very much appreciated research visits to other laboratories. Overall, their installation as full members of research groups for the duration of their visits which included much face-to-face supervision from the host academic led to an increase in the feelings of equity experienced by those students. Many found the visits inspirational and led them to apply for their own UK–Africa funding in the form of fellowships as they saw how effective the arrangement can be in establishing equity in the quality of the research performed.

The delivery of materials science capital equipment to African institutions was also a success. We have delivered X-ray diffractometers to both KNUST and UY; these are considered workhorse instruments in Europe, Asia and USA, mostly all science departments have one, yet these are lacking at these Sub-Saharan institutions and as such represent a real game-changing moment for these departments achieving equity in their research capability with better resourced areas globally. Not only this but we have also made UNIZULU world leading in *in situ* analysis of precursor breakdown with the delivery of the microwave–Raman system (only nine worldwide). Hence, we have more than achieved equity here; we have gone beyond and supplied the tools to make this institution a potential leader in the field worldwide in the next 5–10 years. There has also been added value to this approach; as the reputation of partners in materials science increased throughout the project it meant that more funding for capital equipment could be leveraged outside the programme. An example of this was at UNIZULU: the department acquired an F200 HRTEM microscope valued at R23 million (*ca* £1 million) in 2022. The microscope is now fully functional and forms a major thrust in research in functional materials in the department.

A lesson learned is that significant budget was assigned for travelling in the ACBI proposal and much less on equipment. One of the challenges of researchers in the developing world is having access to proper research facilities (equipment, reagent analysis, etc.). The aspect of equipment purchase in future programmes for capacity strengthening should be considered. However, this was partially addressed in the programme by the lifting of the spend cap on the equipment line which allowed the purchase of several pieces of capital equipment (>£50 000). We have shown that we can effectively deliver game-changing pieces of equipment to these institutions and as such there is no reason why this should not be considered as an effective route to capacity strengthening on research infrastructure in future programmes. It is also important to get buy-in from the management of African institutions. This will ensure that the equipment will have technical support, i.e. a dedicated technician and to ensure that all service and maintenance costs are covered in coming years. A note of commendation here is to be made to the Royal Society—they understood completely the rapidly evolving situation with the pandemic, and it was only through their foresight and flexibility that this aspect of the programme, which has led to a lasting impact in research infrastructure for the African institutions, possible. This highlights the importance of funders of these sorts of programmes building in flexibility into them going forward.

A few new partnerships with industry were established over the period of the award that continue to the present. For example, several new proposals were produced during the period of the grant that used research arising from the ACBI programme. For example, a £10 million bid on the EU green deal was supported by BP plc that proposed the principles of the chemistry in the programme be used to produce electrocatalysts for water splitting. Lewis was involved in an EPSRC grant on hybrid perovskite materials related to solar energy generation that has Johnson Matthey as a partner organization. Revaprasadu engaged with SASOL regarding green hydrogen projects. He later received a grant to the value of R1 million per year for 5 years on a new SASOL/NRF programme. Ndifon developed some local collaboration on solar energy applications with the Renewable Energy research programme of Cameroon’s Institute of Geological and Mining Research, one of the institutes of the Ministry of Scientific Research and Innovation. One joint seminar was engaged: the Institute of Geological and Mining Research (IRGM) of the Ministry of Scientific Research and Innovation and Cameroon’s Rural Electrification Agency (AER) based in Yaoundé, Cameroon. Working visits to these structures enabled the postgraduate students to gain some insight on the applications of materials for their programmes. At IRGM, their renewable energy research programme includes solar energy generation which is still being developed. At AER, rural electrification is expected to be boosted when the Agency embraces the use of solar energy for rural electrification. There is potential for future collaboration and engagement with both IRGM and AER in Cameroon in future owing to the ACBI programme. Awudza has been working with the Royal Society of Chemistry and Pan African Chemistry Network in the capacity building of African chemists in GC–MS and LC–MS techniques. He is also currently working on the development of catalysts for the pyrolysis of waste plastics into fuel (renewable energy). This latter work is under the KNUST’s plastic waste management programme and is in collaboration with Ghana National Plastics Wastes consortium with support from the Ministry of Environment, Science Technology and Innovation, World Bank and UNDP.

## 8. Summary, conclusions and future outlook

Overall, we delivered a comprehensive training programme in materials chemistry to our students. They learned new chemistry and materials science, often at the forefront of the field, and have had the chance to present work and network with eminent research leaders from Europe and the USA at the conferences and training events. They have also had the chance to publish their work in leading outlets. They have also had the opportunity of networking with other early-career chemistry graduates. These networks will be of immense benefit to them in their careers, of which some have already embarked.

A significant highlight for the programme was the repurposing of travel funds that may have been lost owing to COVID for the installation of equipment at the African institutions that will genuinely be game changing (X-ray diffractometers to Ghana and Cameroon, and ICP and microwave Raman systems to South Africa). These will encode for future capacity building on both the postgraduate research and undergraduate training fronts and will provide lasting impact as a legacy of the programme. The programme has also enhanced the working relationship between the four partners.

The project highlighted to us the importance of equitable research. Topics should be vetted carefully to ensure that they are relevant to resource-poor nations. Independent ethics advisors should deliver training on the programmes tailored to consortia. Care should be taken to look at project design and so that it is conducted in an equitable way that is sensitive to cultures. Additionally, projects should also take into account fragile situations, e.g. political instability, civil unrest, that could lead to inequitable situations and write this into risk registers with clear contingency plans. In the future, it might also be useful to arrange for some students/postdocs from the endowed institutions to visit the less endowed ones to experience the conditions under which their counterparts who visit their institutions work. This could help to leverage any attitudes that point to thinking that their visitors are underperforming.

The development of a comprehensive capacity-strengthening plan during the project will leave a legacy and roadmap by which African institutions can improve the quality of their PhD programmes to the level potentially of that rivalling European programmes. However, realistically speaking there would need to be a united push to implement these plans as they detail a significant amount of work and change across institutions that may not be feasible within the lifetime of many of the consortium members. One of the concerns is of course around the equipment that was purchased and the lifetime thereof. Unfortunately, it was not possible with the limited funds available to purchase lasting service contracts for the equipment, so it will be interesting to see how the institutions support the maintenance of the infrastructure beyond the lifetime of the grant. It is hoped that university management will see the value in these pieces of kit in generating excellent research outputs and therefore have a positive attitude towards upkeep and investment.

The consortium members will maintain contact with each other and look for other opportunities for funding UK–African networks. This in part has already started to happen; in 2021, some consortium members (Lewis/Awudza/Ndifon) applied for a large EU Green deal grant addressing local fuel vector storage in Africa but were unsucessful in this endeavour. Awudza is still in contact with some of the consortium members for the development of other proposals aimed at helping with irrigation in Ghana. Although this grant application was unsuccessful it demonstrates the commitment of the partners to continue collaboration. Manchester will receive students from African partners who wish to visit on travel awards. There are also plans for African-based students to move between the African institutions. UNIZULU has become a hub of materials excellence in South Africa. Students from African partner countries will be given an opportunity to visit on new travel-based grants. We are also looking for opportunities for our graduates and students to move between Ghana, Cameroon and South Africa in order to learn first-hand the problems encountered in our respective countries with respect to cutting-edge research and how we can solve them together. With the lessons learned during the ACBI programme we are confident that we can do this.

## Data Availability

This article has no additional data.
